# Primary and acquired resistance to first-line therapy for clear cell renal cell carcinoma

**DOI:** 10.20517/cdr.2023.33

**Published:** 2023-08-02

**Authors:** Serena Astore, Giulia Baciarello, Linda Cerbone, Fabio Calabrò

**Affiliations:** ^1^Medical Oncology, San Camillo Forlanini Hospital, Rome 00152, Italy.; ^2^Medical Oncology, IRCSS, National Cancer Institute Regina Elena, Rome 00128, Italy.

**Keywords:** Renal cell carcinoma, resistance, tumor microenvironment, checkpoint inhibitors, target therapy

## Abstract

The introduction of first-line combinations had improved the outcomes for metastatic renal cell carcinoma (mRCC) compared to sunitinib. However, some patients either have inherent resistance or develop resistance as a result of the treatment. Depending on the kind of therapy employed, many factors underlie resistance to systemic therapy. Angiogenesis and the tumor immune microenvironment (TIME), nevertheless, are inextricably linked. Although angiogenesis and the manipulation of the tumor microenvironment are linked to hypoxia, which emerges as a hallmark of renal cell carcinoma (RCC) pathogenesis, it is only one of the potential elements involved in the distinctive intra- and inter-tumor heterogeneity of RCC that is still dynamic. We may be able to more correctly predict therapy response and comprehend the mechanisms underlying primary or acquired resistance by integrating tumor genetic and immunological markers. In order to provide tools for patient selection and to generate hypotheses for the development of new strategies to overcome resistance, we reviewed the most recent research on the mechanisms of primary and acquired resistance to immune checkpoint inhibitors (ICIs) and tyrosine kinase inhibitors (TKIs) that target the vascular endothelial growth factor receptor (VEGFR).We can choose patients’ treatments and cancer preventive strategies using an evolutionary approach thanks to the few evolutionary trajectories that characterize ccRCC.

## INTRODUCTION

The recent approval of the new first-line combinations, which include immune checkpoint inhibitors (ICIs) both plus VEGFR-TKIs or the anti-cytotoxic T lymphocyte antigen-4 (CTLA-4) monoclonal antibody (mAb), ipilimumab, has revolutionized the treatment of metastatic renal cell carcinoma, reporting improved outcomes in pivotal studies^[1-7]^. Despite the excellent response rates, some patients are either innately resistant to therapy or eventually develop later resistance to it. Hence, a better understanding of the mechanisms underlying VEGFR-TKI and/or ICI resistance will be helpful in selecting patients who might not respond to this kind of approach and developing strategies to overcome resistance.

Today, the only validated risk assessment tool is risk stratification according to the International mRCC Database Consortium (IMDC) score, which is based upon six clinical and laboratory features^[8,9]^. However, this approach lacks the ability to recognize genetic and intrinsic factors that potentially direct response to immunotherapy and has only been thoroughly validated for patients treated with single agent VEGFR-targeted therapies.

Here, we reviewed the most recent research on the factors that contribute to both primary and acquired resistance to VEGF-TKI and ICI with the aim of supplying tools for patient selection and generating hypotheses in an effort to decrease the proportion of patients who do not respond or to postpone the emergence of resistance.

## MOLECULAR SUBSETS IN METASTATIC RENAL CELL CARCINOMA

Clear cell renal cell carcinoma (ccRCC) is a highly inflamed and immune-infiltrated tumor type with high expression of immune checkpoints, such as PD-L1 and CTLA-4. However, ccRCC has the peculiarity of having a high degree of infiltration by exhausted CD8+ tumor-infiltrating lymphocytes (TILs), immunosuppressive cells such as M2-like tumor-associated macrophages (TAMs), regulatory T cells (Tregs), and myeloid-derived suppressor cells (MDSCs), which characterized a tumor microenvironment with immunosuppressive properties^[10-12]^.

Immune cells (IC) are components of tumor immune microenvironment (TIME) and play an important role in modulating immune response to tumor cells^[13]^. These cells could have been implicated both in immune tumor suppression and tumor escape.

More than just identifying individual cells, the microenvironment’s composition could provide insight into the mechanisms causing immune escape, and choosing more effective targets could lead to better results. Using ccRCC samples, Chevrier *et al.* examined the TIME’s composition and discovered a particular exhausted CD8+/PD-1+ T cell phenotype that was defined by the co-expression of inhibitory receptors and might be the cause of immune suppression. Additionally, they discovered CD38 to be a marker of exhaustion in the CD8+/PD-1+ T cell phenotype, and these cells were closely associated with the presence of regulatory CD4+ T cells and of a cluster of macrophages with the highest expression of CD38 and immune suppressive activity.

Rather than focusing on each cell individually, TIME composition and the number of immune cells may be able to predict outcomes more accurately^[14]^.

Additionally, the TIME could be altered by the use of VEGFR-TKIs and ICIs^[15-21]^, and the TIME may also be impacted by genetic changes in ccRCC, such as von Hippel-Lindau (VHL) and PBRM1mutations. As a result, different genomic signatures may confer a different response to a specific treatment. Therefore, a deeper comprehension of the molecular traits that uniquely distinguish ccRCC is required to enhance patient selection, risk stratification, and resistance mechanism definition.

Three major gene expression signatures have been identified using data from the first-line pivotal trial in mRCC (IMmotion 150, JAVELIN RENAL 101, Checkmate 214): Angiogenesis, T-effector (Teff)/IFN-γ response, and myeloid inflammatory gene expression signatures^[22-24]^.

Gene expression patterns that had been previously described in relation to their corresponding biology were used for the definition of gene signatures. Angio: VEGFA, KDR, ESM1, PECAM1, ANGPTL4, and CD34; Teff: CD8A, EOMES, PRF1, IFNG, and CD274; myeloid inflammation: IL-6, CXCL1, CXCL2, CXCL3, CXCL8, and PTGS2^[25-27]^.

Angio gene profile identified a group of patients who would respond to a VEGFR-TKI alone, resulting in superior outcomes with sunitinib in each study. Given that a high T effector gene signature may indicate an improved response to a VEGFR-TKI and ICI combination therapy, the presence of a myeloid signature in the T effector high group identified tumors that are resistant to immunotherapy when used alone, as demonstrated by the worse outcomes for patients treated with atezolizumab alone in the IMmotion 150 trial. Furthermore, a myeloid infiltrate, which is indicative of innate resistance to immunotherapy alone, could be overcome by the addition of a therapy targeting angiogenesis. Gene signatures based on a single class of genes may be effective tools that support patient selection. However, when using two immune checkpoint inhibitors, a combination of gene signatures including both innate and adaptative immune response components may be more suggestive of how TIME and therapies interact and may be more predictive of results. The “Renal 101 Immune signature”, a 26-gene subset of the gene expression signature (GES) that included regulators of innate and adaptive immunological responses (T cell and NK cell), cell trafficking, and inflammation, identified in JAVELIN RENAL 101, is an even more comprehensive molecular predictive tool, underlying the significance of CD8+ T cells in inducing immune response^[23]^.

Table 1 schematically summarizes the correlation of GES (Angio, Teff, Myeloid) with positive outcomes in IMmotion 150, Javelin RENAL 101, and Checkmate 214.

**Table 1:** Correlation between GES with favorable outcomes

	**IMmotion 150^[22]^**			**Javelin renal 101^[23]^**		**Checkmate 214^[24]^**	
	Atezo + Beva	Atezo	Sun	Ave + Axi	Sun	Nivo + Ipi	Sun
Angio^high^			**√**		**√**		**√**
Angio^low^	**√**						
Teff^high^	**√**			**√**			
Teff^low^			**√**				
Myeloid^low^	**√**	**√**					
Myeloid^high^			**√**				
Teff^high^ Myeloid^high^	**√**						
Teff^high^ Myeoid^low^		**√**				**√**	
26-gene immune signature				**√**			
26-gene immune signature					**√**		

Angio: Angiogenic; Atezo: atezolizumab; Ave: avelumab; Axi: axitinib; Beva: bevacizumab; GES: gene expression signature; Ipi: ipilimumab; Nivo: nivolumab; Sun: sunitinib; Teff: T-effector.

## MOLECULAR SUBSETS IN RCC AS BIOMARKER STRATEGIES FOR PERSONALIZED TREATMENT

In 2020, McDermott *et al.* completed a significant research effort to evaluate the outcomes of patients who received a combination of checkpoint inhibitors, applying the previously developed IMmotion 150 signatures to the IMmotion 151 trial^[22]^. In order to develop a new molecular categorization of RCC, they performed an integrative multi-omics analysis of 823 RCC tumors^[28,29]^.

Non-negative matrix factorization (NMF) was used to identify seven distinct molecular clusters, and the distribution of these clusters across IMDC risk groups was assessed. They examined the somatic alterations within each cluster and investigated the clinical outcomes of patients who received atezolizumab in combination with bevacizumab, sunitinib, and atezolizumab across clusters.

Table 2 synthetizes cluster characteristics, gene profile expression, and their correlation with outcomes.

**Table 2:** Molecular clusters by NMF, gene expression profiles, DNA alterations, and correlation with outcomes

	**Cluster 1**		**Cluster 2**		**Cluster 3**		**Cluster 4**		**Cluster 5**		**Cluster 6**		**Cluster 7**	
Name	Angiogenic/ Stromal		Angiogenic		Complement/ -Oxidation		Teff/ Proliferative		Proliferative		Stromal/ Proliferative		snoRNA	
Transcriptional Pathways	Angiogenesis Stroma		Angiogenesis Catabolic metabolism (FAO)		Complement cascade -oxidation		Cell-cycle Teff Anabolic metabolism (FAS)		Cell-cycle Anabolic metabolism (FAS) Myeloid inflammation		Cell-cycle Stroma		snoRNAs	
Gene expression module	TGF, WNT, Hedgehog, NOTCH Stroma-genes: Fibroblast-derived genes *FAP, FN1, PSTN, MMP2*		TGF, WNT, Hedgehog, NOTCH Catabolic mb: *FAO/AMPK* genes *Moderate expression:* *Teff genes*		Complement cascade Cytochrome P450 family *Moderate expression:* *Cell-cycle genes*		Cell-cycle Anabolic mb FAS Pentose phosphate Teff JAK/STAT IFN-α and -γ		Cell-cycle Anabolic mb Myeloid inflammation genes		Cell-cycle Anabolic mb Stroma-genes EMT transcriptional Myeloid inflammation genes		C/D box snoRNAs (SNORDs)	
DNA alterations	PBRM1 VHL KDM5C PTEN		VHL PBRM1 KDM5C		VHL PBRM1 KDM5C PTEN BAP1		VHL CDKN2A/B BAP1		CDKN2A/B TP53 TFE fusions (mTORC1 pathway)		VHL CDKN2A/B TP53		VHL SETD2 PTEN	
PFS Months HR (95%CI)	Ate/bev	Sun	Ate/bev	Sun	Ate/bev	Sun	Ate/bev	Sun	Ate/bev	Sun	Ate/bev	Sun	Ate/bev	Sun
	15.3	13.9	13.8	14.2	8.1	7.1	10.9	6.1	8.3	4.3	6.8	5.2	NR	7.4
	HR 1.11 (0.65-1.88) *P* = 0.708		HR 1.16 (0.82-1.63) *P* = 0.397		HR 0.92 (0.63-1.34) *P* = 0.666		HR 0.52 (0.33-0.82) *P* = 0.005		HR 0.47 (0.27-0.82) *P* = 0.007		HR 0.81 (0.52-1.25) *P* = 0.331		HR 0.10 (0.01-0.77) *P* = 0.028	
OS Months HR (95%CI)	Ate/bev	Sun	Ate/bev	Sun	Ate/bev	Sun	Ate/bev	Sun	Ate/bev	Sun	Ate/bev	Sun	Ate/bev	Sun
	NR	48.2	46.2	NR	35	36.6	38.7	23.3	21.7	15.5	15.9	12.7	NR	NR
	HR 0.94 (0.52-1.72)		HR 1.32 (0.91-1.91)		HR 0.99 (0.64-1.54)		HR 0.66 (0.41-1.06)		HR 0.66 (0.39-1.12)		HR 0.90 (0.57-1.40)		HR NC	

AMPK: Activate protein kinase; Ate: atezolizumab; Bev: bevacizumab; FAO: fatty acid oxidation; FAS: fatty acid synthesis; Mb: metabolism; Sun: sunitinib; TGF: tumor growth factor beta.

Angiogenic clusters (1 and 2) were enriched in the favorable risk group (evaluated both according to MSKCC and IMDC risk categories) and showed better progressive-free survival (PFS). However, angiogenic signature did not differentiate outcomes between arms. No correlation was also seen for patients belonging to cluster 3 (complement/-oxidation).

Otherwise, the poor-risk group was more likely to have proliferative clusters (4-6), with stromal/proliferative clusters demonstrating the shorter PFS irrespective of treatment arm. Teff/proliferative and proliferative cluster results, as well as the snoRNAs cluster, experienced better outcomes with the atezolizumab plus bevacizumab combination.

Among somatic alterations, PBRM1 mutations gave superior results, irrespective of the treatment arm. But in PBRM1 mutant patients, Atezolizumab + Bevacizumab showed improved PFS and ORR than sunitinib. Conversely, CDKN2A/B alterations identified patients with worse prognoses. However, CDKN2A/A-alterated tumors had better PFS and ORR in the atezolizumab + bevacizumab arm compared with the sunitinib arm.

Better outcomes with the combination were also seen for tumors harboring loss-of-function mutations of ARID1A and/or KMT2C^[28]^.

In conclusion, these molecular subsets constitute a novel method of reproducible response prediction that may be useful in patient selection. As shown for clusters 1 and 2, the angiogenesis pathway confers a biological behavior that is comparable for tumors treated with a VEGFR-TKI alone or in combination. A proliferative pattern, on the other hand, suggests a lack of response to a therapy that targets angiogenesis alone and a potentially better response to a combination therapy that includes ICIs. This classification does not include the combination of dual checkpoint inhibitors, which would restrict its reproducibility. However, we are aware that nivolumab + ipilimumab has shown superior outcomes in the category of intermediate-poor risk.

The tumors from patients with favorable risk in this study exhibited a higher expression of the VEGF pathway-associated angiogenesis signature, which provides a potential explanation for why the dual combination failed to improve outcomes in the favorable risk subgroup.

## MECHANISMS OF PRIMARY RESISTANCE TO TYROSINE KINASE INHIBITOR IN RENAL CELL CARCINOMA

Primary resistance is characterized as a lack of response to therapy, which may be caused by the absence of a particular target’s expression or by the existence of inherently resistant clones that do not respond to target therapy. Furthermore, primary resistance may be influenced by spatial and temporal heterogeneity^[30,31]^.

### Primary resistance to VEGF-TKIs

#### Hypoxia and von hippel lindau pathway

The tumor suppressor protein von Hippel Lindau (VHL) is frequently mutated in hereditary RCC. VHL is a target of hypoxia-inducible factors (HIFs), dimeric proteins composed of O2-sensitive subunits (HIF-1, -2 or 3) and a subunit (HIF-2). In the presence of oxygen, these factors facilitated its degradation. VHL inactivation creates a pseudo-hypoxic state and HIF dimers can bind to hypoxia response elements (HREs) to induce angiogenesis and cancer cell proliferation. VHL disease is characterized by a decreased expression of HIF-1 and an increased expression of HIF-2, the latter connected with c-Myc activity^[32]^.

Gordan *et al*. analyzed 160 tumor samples and found that VHL-deficient ccRCCs can be distinguished based on HIF- expression. Three different subgroups have been defined: (1) Wild-type VHL tumors with no HIF- expression; (2) VHL deficient tumors with HIF-1 and-2 expression; and (3) VHL deficient tumors with only HIF-2. The third subgroup (HIF-2 expression) displayed enhanced c-Myc activity and higher rates of proliferation. The authors also demonstrated an interplay between HIF-2, c-Myc and genome instability. Indeed, the VHL-deficient subgroup expressing HIF-2 was even characterized by an upregulation of the homologous recombination (HR) effectors BRCA1 and BARD1, and consequentially, HIF-2 tumors and no HIF-1 ones had a greater ability to repair DNA damage accumulation induced by replication stress^[32,33]^.

Given that VHL-deficient tumors with only HIF-2 expression experienced primary resistance to angiogenesis inhibition, thus HIF-2a alone may identify a subset of RCCs in which targeted therapies lack efficacy.

#### Membrane transporters and lysosomal sequestration

The uptake and efflux of several TKIs (e.g., sunitinib, cabozantinib, pazopanib) could be mediated by multidrug resistance (MDR)-related solute carrier (SLC) and ATB-binding cassette (ABC) transporters, respectively^[34,35]^. A number of variables, including pH, drug concentration, and affinity, can affect the interaction between TKIs and transporters. As a result of insufficient intracellular drug concentration, these transporters can be responsible for intrinsic resistance.

The most studied ABC transporters implicated in MDR include P-glycoprotein (Pgp, ABCB1), multidrug resistance protein 1 (MRP1, ABCC1), and breast cancer resistance protein (BCRP, ABCG2). Due to a substrate-like characteristic, TKIs can be pumped outside the cells with an efflux mechanism at lower concentrations. However, at higher concentrations, TKIs can act as inhibitors.

TKIs usually inhibit ABC transporters without altering their expression or localization. As an example, cabozantinib competitively interacts with the drug-substrate binding site to decrease the ATPase activity of the ABCG2 transporter^[36]^. Pazopanib has both substrate-like and inhibitory effects. In the canine kidney cell line MDCKII, it was reported as both an ABCB1 and ABCG2 substrate and as an inhibitor of ABCB1 and its efflux characteristics^[37-39]^.

Lysosomal sequestration is another mechanism of resistance based on drug physicochemical properties. This phenomenon is related to ABC transporters since these pumps are present on the membranes of intracellular compartments and regulate the drug’s influx into lysosomes^[37,40,41]^. Lysosomal sequestration has been shown to impact the effectiveness of the drugs sunitinib and pazopanib^[42]^.

Lysosomal intake-induced resistance can be reversed. Indeed, after removing sunitinib from tumor cell culture, cell lysosomal capacity was restored, regaining drug sensitivity. This can give an explanation of the recovered sensibility to sunitinib experienced by patients after treatment interruption and subsequent rechallenge^[37,42,43]^.

## SECONDARY AND ACQUIRED RESISTANCE TO VEGFR-TKIs

**Figure 1:**
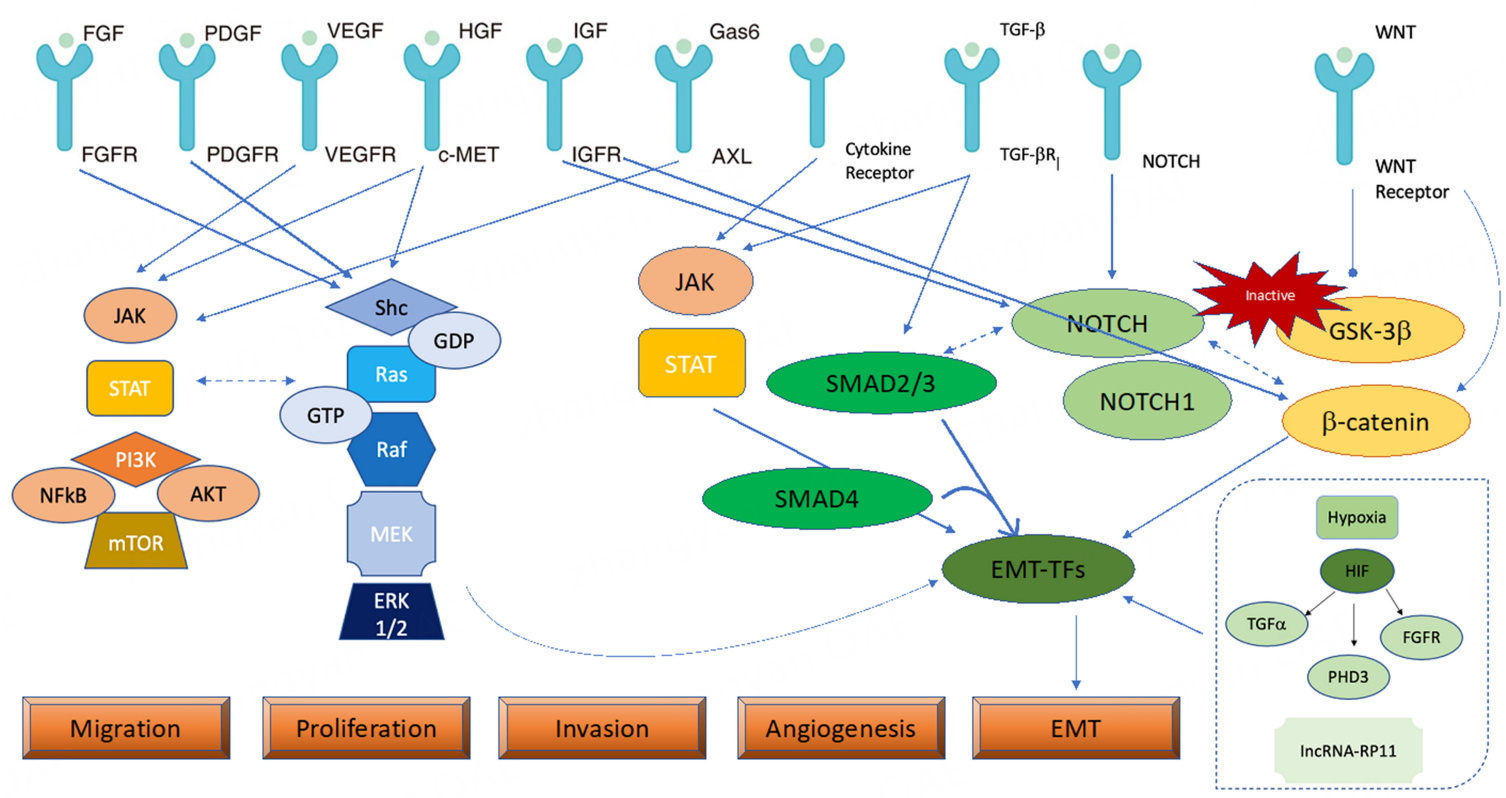
Cross-talking between TKI receptors, hypoxia and growth factor receptors and relationship with tumor growth and resistance to TKIs. Several trans-membrane TKI receptors interact with each other and mediate the activation of shared pathways, most implicated in tumor growth and angiogenesis. TGFβ/Smad pathway and the interaction of some cytokines, such as IL-6 with their receptors, together with the Notch signaling pathway and wnt/β-catenin pathway induce EMT transcription factors and EMT as a mechanism of resistance to the inhibition of angiogenesis. Finally, hypoxia promotes both angiogenesis and EMT. AKT: Protein kinase B; AXL: AXL Receptor; EMT: epithelial mesenchymal transition. EMT-TFs: epithelial mesenchymal transition transcription factors; Erk1/2: elk-related tyrosine kinase; FGF: fibroblast growth factor; FGFR: fibroblast growth factor receptor; Gas6: growth arrest specific protein-6; GSK-3: glycogen synthase kinase 3 beta; HGF: hepatocyte growth factor; HIF: hypoxia-inducible factor; IGF: insulin growth factor; TGF-: tumor growth factor; IGFR: insulin growth factor receptor; JAK: janus kinase; Mek1/2: MAP kinase-ERK kinase; MET: hepatocyte growth factor receptor; mTOR: mammalian target of rapamycin; NFκB: nuclear factor kappa B; PDGFR: platelet derived growth factor receptor; PDGF-β: platelet derived growth factor-β; PI3K: phosphatidyl inositol 3-kinase; Raf: RAF proto-oncogene serine/threonine-protein kinase; Ras: rat sarcoma protein; STAT3: signal transducer and activator of transcription 3; VEGF: vascular endothelial growth factor; VEGFR: vascular endothelial growth factor receptor; VHL: von hippel-lindau protein.

Bergers and Hanahan categorize resistance to VEGFR-TKI as intrinsic or primary and adaptative or evasive (secondary)^[44]^. Sometimes, it was impossible to clearly and immediately shift the biological basis of these two types of resistance. The majority of the time, primary resistance could be explained by the abundance of angiogenic receptors and downstream pathways. However, the hypoxic state induced by VEGFR-TKI therapy could be responsible for an “angiogenic switch” towards a different molecular pathway, driving a different pattern of response [Figure 1].

Acquired resistance develops throughout treatment, usually following an initial response to therapy, and could be induced by the pressure of a specific therapy. The selection of particular clones that are resistant to therapy results in the progression of cancer. Additionally, cancers might develop resistance to a particular treatment through other pathways that are not affected by targeted therapy.

### Tumor plasticity

A loss of cell polarity and contact promotes epithelial-mesenchymal transition (EMT). E-cadherin and other epithelial cell markers are downregulated during this transition, while mesenchymal markers including N-cadherin, vimentin, fibronectin, different matrix metalloproteases (MMPs), and 1 and 3 integrins are upregulated^[45,46]^. It has been shown that HIF-1 activation caused by hypoxia can induce EMT, a process that is associated with drug resistance^[47-51]^. EMT-associated transcription factors (EMT-TFs), such as TWIST1, ZEB1, or SNAI1, can be stimulated to express themselves directly or indirectly by the stabilization of HIFs under hypoxia. TGF/TGFR, NF-B, and NOTCH signaling are the three regulatory mechanisms most extensively studied for their potential role in triggering EMT^[52-55]^.

Insulin-growth factor (IGF) signaling, which interacts with the NOTCH and Wnt/-catenin pathways, has been shown to be a modulator of EMT^[56]^.

Sharma *et al.* provided evidence that sunitinib-treated RCC tumors underwent a mesenchymal transformation as seen by higher expression of N cadherin and decreased expression of E cadherin, which were both connected with an elevation of TGF and IGF1R. Patients with mRCC who expressed IGF1R and TGF and subsequently had EMT had worse outcomes, as shown by data from the Cancer Genome Atlas (TCGA) database^[57]^.

Hwang *et al.* analyzed gene expression profiles and copy number variations of 10 metastatic ccRCC tumor samples before treatment and immediately after disease progression to a TKI. Microarray analysis of pre- and post-treatment ccRCC tumors demonstrated an increased expression of EMT-related genes including CD44, SNAI2, TWIST, and CLDN1 in TKI-resistant cells, acquiring migration and invasion capacity. In this study, the authors demonstrated that CD44 depletion significantly decreased cell invasiveness. Claudin-1 is a component of tight junctions, and it has been suggested that it could be implicated in EMT induction. In this study, claudin-1 expression seems to be a mediator of TKI resistance in both clinical and experimental models. Furthermore, compared to pre-treatment ccRCC, TKI-resistant tumors had an increased proportion of sarcomatoid features^[58]^.

It may be crucial to understand the various pathways that underlie EMT and the indicators of this process in order to design strategies that combine therapies addressing both hypoxia and EMT.

### Non-angiogenic pathway and by-pass pathways

Vascular co-option is a way to use pre-existing vessels. Using 164 lung metastasis specimens, Bridgeman *et al*. identified four different histopathological growth patterns (HGPs), each with a different vascularization (alveolar, interstitial, perivascular cuffing, and pushing). The tumors vascularize through angiogenesis exclusively in the pushing HGP pattern; a co-option vascular mechanism was employed in the other patterns. The authors showed that vascular co-option might act as a mediator of sunitinib resistance. In mice models, sunitinib induced a switch from the most frequent angiogenic pushing HGP to an alveolar/interstitial HGP that vascularizes through vessel co‐option, thus inducing resistance^[59]^.

Furthermore, cancer resistance can arise when tumor cells employ other signaling pathways that are unaffected by VEGF/VEGFR suppression.

#### Fibroblast growth factor/fibroblast growth factor receptor pathway

Fibroblast growth factors (FGFs) are proteins that are involved in proliferation, differentiation, migration, and apoptosis of tumor cells^[60]^.

FGF2, also known as basic FGF, has been recognized as a potential mediator of TKI resistance. Cell cultures were treated with VEGF and 100 nM sunitinib by Welti *et al.* Endothelial cell proliferation was restored by FGF1 and FGF2 to levels that were comparable to (FGF1) or greater than (FGF2) those seen in the absence of sunitinib^[61]^. Indeed, FGF2 induces angiogenesis through the activation of signaling pathways such as Ras-Raf-MEK-ERK 1/2 and PLC-PKC, bypassing the VEGF/VEGFR signal. It has been reported that FGF2 upregulates the expression of both fibroblast growth factor receptor (FGFR) and VEGFR in endothelial cells and systemic administration of VEGFR-2 antagonists inhibits both VEGF and FGF2-induced angiogenesis in vitro and in vivo^[62-64]^. In contrast to what was expected by previous studies, Welti *et al*. showed that while sunitinib inhibits VEGFR2-mediated activation of ERK 1/2 and PLC, it is not able to prevent the FGF2-mediated activation of these pathways, raising the possibility that cancer cells may use this way to bypass VEGF-mediated angiogenesis inhibition^[61]^.

#### MET/HGF signaling

When the hepatocyte growth factor/scatter factor (HGF/SF) binds to its receptor tyrosine kinase MET, the activation of the RAS-MAPK and PI3K-AKT pathways results in the development and angiogenesis of endothelial cells. HGF/SF-MET interaction is a potent regulator of the angiogenic switch. Common signaling intermediaries such as ERK-MAPK, protein kinase B (AKT), and focal adhesion kinase (FAK) are activated by both MET and VEGFR^[65-68]^.

A remarkable new finding is that the MET/HGF pathway may be activated by hypoxia caused by blocking angiogenesis with VEGFR-TKIs in a HIF-mediated manner, hence increasing the MET-dependent spread of cancer cells^[69-72]^.

Additionally, it is hypothesized a relationship between the MET axis and the immune system. Several immune cells, including mast cells, neutrophils, and dendritic cells (DCs), may have increased MET expression. MET/HGF-SF signaling could impact the ability of T cells to respond competently to cancer cells, by reducing the DCs’ capacity to present antigens and recruiting immunosuppressive cells. Therefore, MET inhibition may be a way to restore neutrophils and DCs’ capacity^[73,74]^.

Cabozantinib is an oral multiple tyrosine kinase receptor inhibitor: VEGFR2, c-MET, and RET. Inhibition of VEGFR and c-MET decreases resistance to VEGFR inhibitors via the c-MET axis. However, resistance to MET inhibition can occur. Huang *et al.* reported some of the most common mechanisms inducing acquired resistance to HGF/MET-target therapy: hypoxia-induced MET phosphorylation reduction, with no effect on downstream signaling pathway, mutations in the MET kinase domain, bypass signaling, copy number changes and constitutive activation of AKT and ERK-MAPK pathway^[73,75-77]^.

#### GAS6/Axl signaling

AXL is a receptor tyrosine kinase (RTK) that is a member of the TAM RTK. AXL signaling is implicated in tumor growth, EMT, angiogenesis, metastasis spread, and the development of resistance to targeted therapy. The AXL-ligand Gas6 is a vitamin K-dependent protein and the GAS6/AXL signaling can be constitutively activated in ccRCC cells^[78-82]^.

Gustafsson *et al.* found that sunitinib enhanced Gas6-induced AXL phosphorylation in ccRCC, and consequentially activated the AKT pathway. Indeed, in the absence of sunitinib, the activation of the main MAPK pathways (ERK1-2, P38MAPK, and SAPK-JNK) by Gas6 alone was insufficient to activate AXL. Furthermore, it was shown that Gas6 activated the EGFR pathway when sunitinib was present. This pathway, along with AXL, is thought to be implicated in cancer’s resistance mechanism^[83]^.

#### Cytokines

Treatment with sunitinib has been shown to increase the expression of IL-6 and IL-8. These cytokines have been linked to TKI resistance, suggesting that they could play an important role in inducing angiogenesis in a HIF-independent way. IL-6 activates the AKT/mTOR and transcription factor STAT3 cascade, resulting in increased expression of VEGFA and VEGFR2. In endothelial cells, IL-8 promotes the accumulation of VEGFA mRNA in normoxic conditions as well as the endothelial cells are exposed to hypoxia. Even when HIF-1 is blocked, CXCL8/IL-8 can still induce VEGFA promoter-driven transcription^[84,85]^.

Pilskog *et al.* evaluated the expression of IL6R in RCC tumor cells and discovered that the expression of IL6Ra may predict responsiveness to TKI treatment. In fact, a significant correlation between IL6R expression and the objective response rate was identified but not with PFS or OS, indicating that IL6R expression may have predictive significance. IL-6 ligand expression may also play a prognostic role, as demonstrated by the association between its lack of expression or low expression with PFS^[86,87]^.

Huang *et al.* developed sunitinib-resistant xenograft models and discovered that sunitinib-treated ccRCC cell lines developed resistance and displayed an elevated IL-8 expression. They observed that only when sunitinib treatment was sustained over a longer period did the reduction of IL-8 function decrease tumor growth. Only after the emergence of resistance to tyrosine kinase inhibition could IL-8 function inhibition affect tumor growth^[88,89]^.

### Tumor microenvironment and immune cells as mediators of TKI-resistance

**Figure 2:**
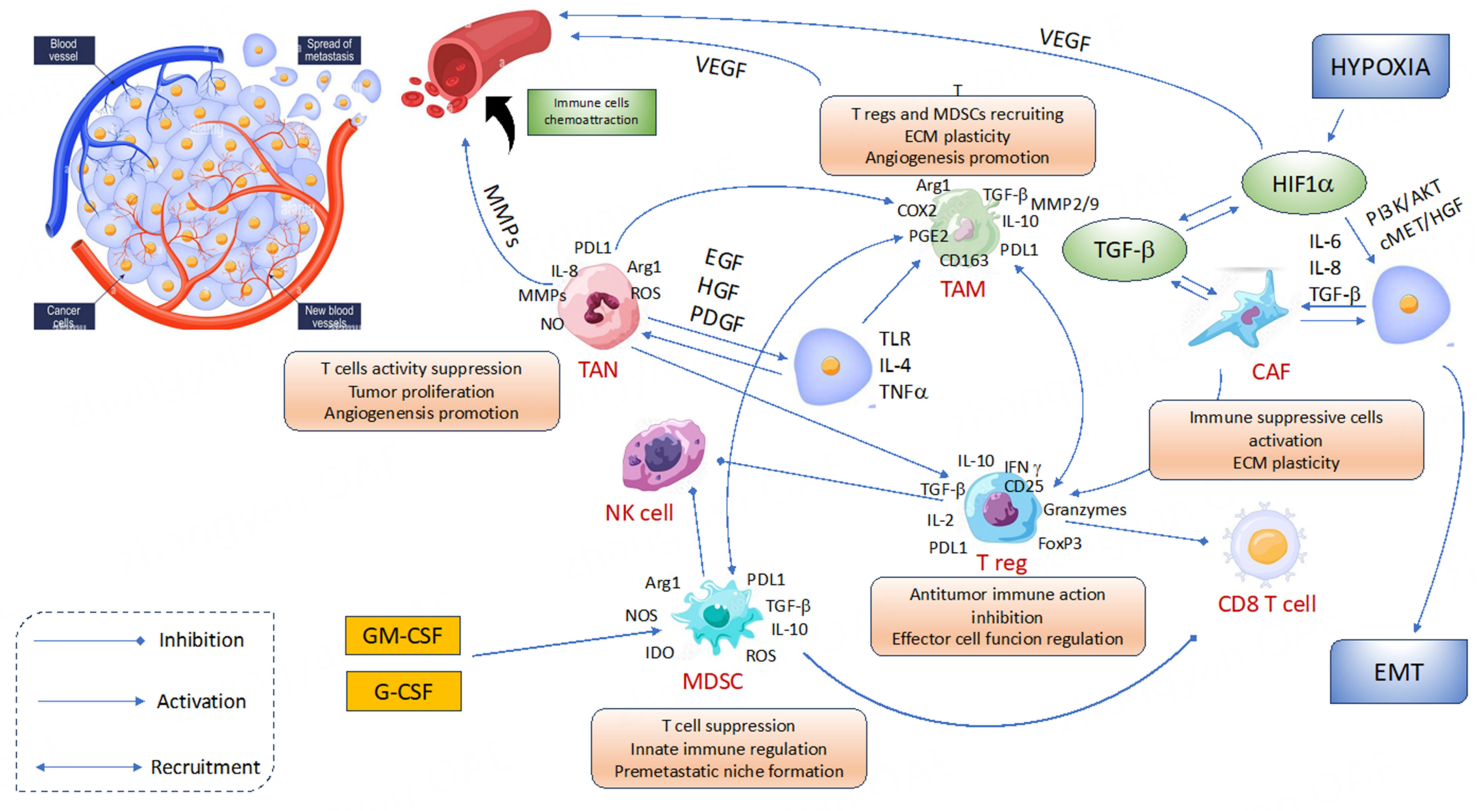
Interaction between angiogenesis and tumor microenvironment. Immune cells are recruited by chemokines and angiogenic factors and the tumor infiltration by immune cells is, in turn, implicated in promoting angiogenesis. The balance between immune response and immunosuppression is crucial to induce tumor killing from one side or tumor escape to the other. CAF: Cancer-associated fibroblasts; EGF: epidermal growth factor; EMT: epithelial-mesenchymal transition; G-CSF: granulocyte colony-stimulating factor; GM-CSF: granulocyte-macrophage colony-stimulating factor; HGF: hepatocyte growth factor; HIF-1α: hypoxia-inducible factor 1α; IFNγ: interferon-γ; IL-2/4/6/10/8: interleukin; MDSC: myeloid-derived suppressor cell; MMP: matrix metalloproteinases; NK: natural killer; PDGF: platelet-derived growth factor; PDL1: programmed death-ligand 1; TAM: tumor-associated macrophages; TAN: tumor-associated neutrophils; TGF-β: tumor growth factor beta; TLR: toll-like receptor; TNFα: tumor necrosis factor α; Treg: regulatory T cells; VEGF: vascular endothelial growth factor^[90]^.

There is growing interest in the connection between TIME, immune cells, and angiogenesis, and there is evidence that the tumor microenvironment has a direct role in the emergence of resistance to targeted therapies. The recently approved combination of ICIs and VEGFR-TKIs is also supported by this association. Therefore, it is critical to understand how TIME affects the mechanisms of resistance to target therapy in order to better understand resistance to combination therapy [Figure 2].

#### MDSCs

MDSCs are the major component of TIME. Growing evidence indicates that tumors release pro-angiogenic signals that recruit MDSCs, which may act as mediators in the development of resistance to TKIs^[91-94]^.

Ko *et al*. had demonstrated that sunitinib therapy significantly reduced MDSC accumulation in tumor-bearing mice models, leading to improved peripheral T-cell function. It appears that even when sunitinib diminishes peripheral MDSC accumulation, intra-tumoral MDSCs can be much less impacted. Intra-tumoral MDSCs from sunitinib-treated mice retained T-cell suppressive capacities comparable to those from untreated mice. The authors quantified MDSC subsets in tumor specimens of untreated and sunitinib-treated RCC. In contrast to the substantial reduction in peripheral blood MDSCs seen in RCC patients treated with sunitinib, the proportion of MDSCs in tumor samples nevertheless remained greater and these cells maintained their suppressive capabilities, as assessed by IFN production.

Additionally, sunitinib did not significantly affect the amount of GM-CSF produced by RCC tumors in vitro. Due to the stimulation of the STAT5 pathway, the pro-proliferative cytokine GM-CSF confers peripheral MDSCs sunitinib resistance. Sunitinib decreased pSTAT3 in the absence of GM-CSF, which made MDSCs more susceptible to the drug. In contrast, STAT5-mediated pathways are prominent in the presence of GM-CSF, resulting in a phenotype of MDSCs that is resistant to sunitinib^[95]^.

These findings have been confirmed by Finke *et al*., who examined MSDCs both in peripheral blood and in tumors of RCC patients. They discovered that the sunitinib-induced maximum drop in MDSC numbers occurred after the second cycle and continued until later than the fourth cycle, although there is some recovery in MDSC levels at this point. In this study, the impact of pro-angiogenic factors on sunitinib resistance was also evaluated. Pro-angiogenic variables’ effects on sunitinib resistance were also assessed in this study. The analysis of tumor tissue lysates from patients who had neoadjuvant therapy revealed that there was an increase in the expression of pro-angiogenic proteins [Matrix metallopeptidase (MMP9), MMP8, and IL-8] in tumors with a greater level of MDSCs. Additionally, sunitinib causes an increase in plasma IL-8 levels, and larger levels are associated with a worse PFS. The presence of MDSCs in tumors might promote the production of IL-8, which could activate alternative pro-angiogenic pathways (MMP9/MMP8/IL8) to prevent cell death^[96]^.

C Marcela Diaz-Montero *et al*. used a patient-derived xenograft (PDX) model of RCC and performed a microarray analysis of sunitinib-responsive and -resistant tumors. Resistance to sunitinib was associated with the upregulation of genes involved in cell movement and immune cell trafficking in both human and murine expression analyses. Furthermore, tumors resistant to sunitinib had higher levels of G-CSF and, consequently, higher levels of G-MDSCs. G-MDSCs are MDSCs that resemble granulocytes both phenotypically and functionally. G-MDSCs have the ability to inhibit the immune response, and they can additionally promote angiogenesis and the spread of tumors^[97]^.

#### Cancer-associated fibroblasts

Tumor-growth factor (TGF) and platelet-derived growth factor (PDGF) induce the transformation of tumor fibroblasts (FHNR) in cancer-associated fibroblasts (CAFs), which play a key role in tumor progression, producing pro-tumoral cytokines (e.g., IL-6, IL-8, TNF, IL-10). Fibroblast-associated protein (FAP) is a marker of CAFs. Ambrosetti *et al*. demonstrated that high levels of FAP mRNA were correlated with shorter PFS and OS in metastatic ccRCC (PFS, *P* = 0.054 and OS, *P* = 0.022). Sunitinib stimulated FHNR to differentiate towards CAFs. Mice with induced mRCC were treated with sunitinib or with placebo. Compared to the placebo group, the sunitinib-treated mice’s tumors had higher FAP mRNA levels (*P* = 0.049). Sunitinib primary resistance has also been linked to CAFs, which create a barrier to the drug’s ability to reach tumor cells. Finally, CAFs appear to be a mediator of tumor cell EMT^[98]^.

#### TAMs

Some of the most numerous immune cells identified within tumors are TAMs. Two subsets of TAMs are typically recognized: M1 and M2. M1-like TAMs have a pro-inflammatory phenotype and inhibit tumor growth, whereas M2-like TAMs have tumor-promoting capabilities involving immuno-suppression, angiogenesis, and neovascularization, as well as stromal activation and remodeling^[99]^. The functional phenotype M2-like is induced by hypoxia of the TIME, resulting in tumor escape^[100]^.

In the genomic and transcriptomic analysis of ccRCC patients treated with TKIs in the COMPARZ phase III trial, Hakimi *et al.* observed significantly worse OS (HR 1.54; 95%CI: 1.17-2.03; *P* = 1.98) among subjects with high macrophage infiltration and higher macrophage infiltration (Kruskal-Wallis test, *P* = 0.02) in patients who experienced progressive disease. Furthermore, they found that a high M2-macrophage infiltration (M2^high^) was associated with poor OS (HR 1.38; 95%CI: 1.06-1.81; *P* = 0.019) and PFS (HR 1.40; 95%CI 1.09-1.78; *P* = 7.90) compared to the M2^low^ group. Depending on the TKI used, macrophage infiltration and its impact on the outcomes differed. TAMs infiltration was a prognostic factor in patients receiving pazopanib but not sunitinib. This supports the notion that sunitinib primarily affects MDSCs^[101]^.

#### TIL and TKIs interaction with immune-cell infiltration

TIL correlated with poorer prognosis and shorter survival in RCC^[102]^. Liu *et al.* compared the percentage of immune cells in TKI-exposed RCC tissue with control samples and they found an increased CD3+ T-lymphocyte infiltration, CD45RO+ T-lymphocyte infiltration, CD4+ T-lymphocyte infiltration and CD8+ T-lymphocyte infiltration both after sunitinib and bevacizumab treatment. The infiltration of CD68+ macrophages was higher in sunitinib-treated versus control RCC specimens (25.2% *vs.* 20.3%, *P* < 0.05) but not in bevacizumab-treated samples (21.3% *vs.* 20.3%, *P* > 0.05). In sunitinib-treated patients, a higher percentage of tumor-infiltrating CD4+ T lymphocytes was associated with shorter OS and PFS. TIL composition might be modulated by VEGF target treatment. Indeed, although not changing either the ratio of CD4+FOXP3+ Tregs to total CD4+ T lymphocytes or the ratio of Treg to CD8+ T lymphocytes, CD4+FOXP3+ Tregs were greater in cases treated with bevacizumab or sunitinib.

Tregs infiltration was higher in sunitinib-treated patients with shorter OS and PFS. Finally, sunitinib directly enhanced PD-L1 expression, and those patients who had higher PD-L1 expression had shorter OS and PFS (*P* < 0.05) after receiving sunitinib treatment^[103]^.

The analysis of T cell subsets and MDSCs in peripheral blood mononuclear cells (PBMCs) from ccRCC patients receiving cabozantinib and other therapies (nivolumab and pazopanib) revealed that T cell subsets composition changed after treatment. Indeed, cabozantinib treatment increased the proportion of Th9, Th22, and Th17 cells while having no effect on the number of Th2 cells, Th1, Treg, and CD8+ T cell populations. Among these T cells, the proportion of Th22, but not Th9, was associated with better outcomes^[104]^.

### Epigenetic modification

#### Non-coding and micro RNA

Non-coding RNAs, known as microRNAs (miRNAs), have significant functions in modulating the expression of genes. The role of miRNAs in resistance to TKIs is still being investigated^[105]^.

Yamaguchi *et al.* conducted one of the first attempts to profile miRNAs in resistant RCC cell lines as they developed two sunitinib-resistant cell lines and performed microarray and RT-qPCR analysis on them. They identified seven miRNAs (miR-575, miR-642b-3p, miR-4430, miR-18a- 5p, miR-29b-1-5p, miR-431-3p, miR-4521) whose expression was linked to sunitinib resistance.

It was reported that miR-4430 had a role in modulating expression genes implicated in the inhibition of PTEN/mammalian target of rapamycin (mTOR) signaling pathway. miR-18a-5p is associated with hypoxia-inducible factor 1 alpha (HIF1A)^[106]^. Sunitinib- resistant cell lines had higher miR-4430 levels and lower miR-18a-5p ones, suggesting that the acquisition of sunitinib resistance was associated with PTEN downregulation, and FGF1 and HIF1A upregulation^[107]^ [Table 3].

**Table 3:** Major determinants of primary and acquired resistance to VEGFR-TKIs

**Primary resistance to VEGF-TKIs**	
Missed targets	VHL-deficient tumors with HIF2 expression^[40,41]^
Insufficient intracellular drug concentration	MDR-related solute carrier (SLC) and ATB binding cassette (ABC)^[44-46]^ Lysosomal sequestration^[47-50]^
**Secondary resistance to VEGF-TKIs**	
Tumor plasticity	Epithelial-mesenchymal transformation (EMT) induced by hypoxia and Insulin-growth factor (IGF)^[54-64]^
Non-angiogenic pathways	Vascular co-option
Bypass pathways	FGF/FGFR: FGF2-mediated activation of ERK 1/2 and PLC pathway is not inhibited by sunitinib^[68]^ MET/HGF: Hypoxia induced by VEGFR-TKIs promotes MET-depended tumor growth^[76-79]^. GAS6/AXL: VEGFR-TKIs treatment enhances the activation of the MAPK pathway by AXL^[90]^.
Tumor microenvironment interactions	Cytokines^[91-93]^ ·IL-6 activates the AKT/mTOR pathway, inducing VEGFA and VEGFR2 expression. ·IL-8 induces VEGFA transcription even when HIF-1 is inhibited. MDSCs^[100-102]^ ·GM-CSF confers sunitinib-resistance to peripheral MDSCs, via the STAT5 pathway. ·MDSCs produce pro-angiogenic proteins. CAFs^[103]^ ·FAP mRNA (a marker of CAFs) levels are correlated with worse outcomes. TAMs^[105-106]^ ·Hypoxia induces M2-macrophages phenotype. ·M2-like TAMs promote tumor growth. TILs^[108]^ ·TKIs treatment induces CD4+ T cells and Tregs infiltration. ·CD4+ T cells and Tregs infiltration correlate with worse outcomes.
Epigenetic modifications	Non-coding RNAs and miRNAs^[116]^ Sunitinib-resistant cells had higher miR-4430 (PTEN/mTOR signaling) and lower miR-18a-5p (FGF1 and HIF1A signaling) levels

CAFs: Cancer-associated fibroblasts; FAP: fibroblast associated protein; FGF: fibroblast growth factor; FGFR: fibroblast growth factor receptor; HIF: hypoxia-inducible factor; HGF: hepatocyte growth factor; MDR: multidrug resistance; MAPK: mitogen-activated protein kinases; MDSC: myeloid-derived suppressor cells; miRNAs: microRNA; TAMs: tumor-associated macrophages; TILs: tumor-infiltrating lymphocytes; VHL: von hippel-lindau.

## PRIMARY RESISTANCE TO ICIs

Availability of antigens and dendritic cells’ (DCs’) presentation of them, T-cell trafficking and tumor infiltration, T-cell effectiveness, and equilibrium between regulatory and cytotoxic cells in the TIME composition are all requirements for an immune response against tumor cells to be successful. Immune escape may occur in any of these phases, resulting in either primary or acquired resistance to immune checkpoint inhibitors^[108]^.

### Antigen availability and DCs presentation capacity

The recognition of a specific antigen by antigen-presentation cells (APCs) is the first step in a successful immune response. Major Histocompatibility Complex (MHC) proteins, which are classed as class I on all nucleated cells or class II on specific immune system cells such as macrophages, dendritic cells, and B cells, are also expressed on the surfaces of APCs. These proteins are necessary for the process of antigen presentation, which activates T cells in response to the antigen and results in a successful immune response^[109]^.

Antigen availability may be influenced by the tumor mutational burden, and the absence of neoantigens may be caused by epigenetic alterations^[110]^. RCC has a relatively low mutational load. De Velasco *et al.* reported a low mutational load in their full exome transcriptome analysis of metastatic RCC patients included in TCGA, with a median of 1.42 mutations/Mb (range: 0.035-2.77). Classifying the 54 patients according to the IMDC risk criteria, no differences were seen in mutational load (*P* = 0.39), as well as in the expression of cytolytic genes - granzyme A (GZMA) and perforin (PRF1) - or in selected immune checkpoint molecules (PD-1, PD-L1, PD-L2, CTLA-4) (*P* > 0.05 for all)^[111]^.

Lack of antigen presentation could be affected by MHC mutations or defects in DCs’ functions and maturation.

Beta-2-microglobulin (B2M), an important subunit of MHC class I, has essential biological functions and roles in tumor immunity. *B2M* gene deficiency and loss of Beta-2-microglobulin, induced by mutations and epigenetic regulation, can lead to complete loss of MHC class I antigen expression. B2M mutations frequently result in MHC defects that cannot be repaired, and immunotherapy is ineffective at restoring MHC expression^[112]^.

The maturation of DCs can be influenced by numerous factors. Hypoxia, which creates an acidic environment with a higher amount of lactates, is one of these. Recent data suggests the existence of DCs with immune-suppressive characteristics. These cells may overexpress pathways involved in immune tolerance: e.g., STAT3 which induces S100A9, a gene that prevents DCs from maturing and recognizing antigens, or FOXO3, a transcription factor that induces the expression of indolamine 2,3-dioxygenase (IDO), arginase, and TGF-β and inhibits co-stimulatory molecules^[113]^.

In the TIME, dendritic cells must already be present. A study conducted by Spranger *et al*. in melanoma murine models revealed that tumor-intrinsic active -catenin signaling results in T-cell exclusion and resistance to ICIs. Tumors with active -catenin exhibited a nearly complete absence of activated T cells, according to an analysis of immunological infiltrates. This absence was attributed to the loss of dendritic cells, particularly CD103+ dendritic cells with diminished IFN-β expression. The number of CD103+ dendritic cells was similarly decreased in the tumor-draining lymph nodes, but not in the spleen, thus reflecting the fact that these DCs were not recruited due to a defect in chemotactic signals, such as CCL4. Furthermore, when treated with dual checkpoint inhibition, no therapeutic effect in reducing tumor growth was detected in active -catenin mice. Re-introduction of dendritic cells restored tumor response to ICI^[114]^.

#### Human endogenous retroviruses

Human endogenous retroviruses (hERVs) are components of about 8% of our genome that possibly originated by incorporating ancestral exogenous viruses. There are only a limited number of tools available for quantifying and identifying hERVs. They could be a source of neoantigen and mediate immune response in TIME. Smith *et al*. identified more than 3,000 transcriptionally active hERVs within the TCGA pan-cancer RNA-Seq database. They explored two mechanisms by which hERV expression could affect the tumor immune microenvironment in ccRCC. The first mechanism involved innate immune response and activation of the RIG-I-like pathway signaling by double-stranded RNAs (dsRNA). The second was related to the adaptive immune response, as documented by the hHERV epitope-trigged activation of the B cells. Patients with both RIG-I–like downregulation and BCR-associated signatures upregulation had significantly shorter overall survival, while those with higher expression of the RIG-I-like signature had longer overall survival. Two significant tumor-specific hERVs were lastly found in ccRCC (CT-RCC hERV-E and hHERV 4700). The CT-RCC hERV-E displayed a Treg signature and was the second most differentially expressed hERV. In an aPD1-treated ccRCC dataset, hERV 4700 (HERVERI/gammaretro-virus-like) expression was higher in tumor samples compared to normal tissue, and it was associated with response to immunotherapy. This could occur because hERV 4700-derived epitopes may be the target of an antitumor response mediated by aPD1 mAb^[115,116]^.

### Immune cell trafficking and recruitment

As discussed above, activation of the WNT/-catenin pathway reduces the recruitment of dendritic cells in TIME, affecting T-cell activation^[114]^.

Given that pre-existing T-cells in the tumor are essential for response to ICIs, various mechanisms that may cause initial resistance to immunotherapy with the end result of the absence of immunologically competent cells in TIME have been investigated so far.

PTEN is a lipid phosphatase that inhibits the activity of PI3K/AKT signaling. PTEN loss has been associated with altered TIME T-cell composition. Peng *et al*. found that a lower CD8+ T cell tumor infiltration was present in melanomas with PTEN loss compared to tumors with PTEN expression (*P* < 0.001). Furthermore, PTEN loss resulted in both an up-regulation of inhibitory cytokines including CCL2 and VEGF and a down-regulation of the T cell effector molecules IFN- and granzyme B. Since PTEN loss and PI3K/AKT pathway signaling both contribute to immunotherapy resistance, it is possible to reverse this effect by targeting the PI3K-AKT pathway^[117]^.

### T-cell activity in TIME and microenvironment composition

T cells get activated when they come into contact with APCs and the MHC complex through the TCR. Following this, T cells mostly develop into cytotoxic T cells (CD8+ cells) or T-helper (Th) cells (CD4+ cells), and they release cytokines that further regulate the immune response^[108]^. Cytotoxic CD8+ T lymphocytes are mainly responsible for killing tumor cells. MHC class II proteins trigger the activation of CD4+ Th cells, which lack the ability to cause cytotoxicity. In fact, they are implicated in the recruitment of additional immune cells, producing cytokines. IFN-γ is a mediator of the Th1 cell response^[109]^.

As tumors grow, persistent exposure to IFN-γ may be responsible for a down-regulation of IFN signaling downstream, resulting in primary immunotherapy resistance. Mutations in IFN receptors, Janus kinases JAK 1/JAK2, are studied mechanisms by which cancer cells could have an advantage in developing resistance to IFN-mediate anti-proliferative effects. Shing *et al*. discovered that loss of function mutations in JAK1/2 in melanoma cells could inhibit T cells killing capacity and IFN’s ability to induce PD-L1 expression, making pharmacological suppression of the PD-L1/PD-1 interaction ineffective. Reduced T-cell trafficking, along with decreased production of chemokines such as CXCL9, CXCL10, and CXCL11, could potentially account for the lack of response^[118]^.

For a competent immune response to be effective, there must be a balance between cells that inhibit immunity and cells that promote response inside the tumor microenvironment. TME is dynamic and constantly changes. The TIME of ccRCC is unusual, and it is distinguished by a strong inflammatory profile and T-cell infiltration. The primary immunosuppressive cells in TIME are TAMs, MDSCs, and regulatory T cells (Treg)^[119]^**.**

#### TAMs

In ccRCC, cancer-cell-derived factors such as IL-1β, IL-6, IL-10, tumor necrosis factor-α, epidermal growth factor, and TGF-β induce macrophage polarization towards a M2 phenotype by cell-cell interactions. CD163+ M2 TAMs contributed to poor clinical prognosis in patients with ccRCC by activating STAT3 pathway. PI(3)Kinase γ (PI3Kγ) is found to be implicated in themacrophage switch from a M1-phenotype to a M2-phenotype, inhibiting Akt and mTOR together with the NFκB and C/EBPβ activation. As a result, the immune suppressive phenotype promotes tumor growth and inflammation^[120,121]^.

#### Tregs

Through the production of IL-10 and TGF-, Tregs can mediate immunosuppression by inhibiting T cells and APCs functions. These cells can be recruited in TIME by chemokines and cytokine produced by exhausted T-cells. Furthermore, cancer cells can play out escape mechanisms upregulating Tregs in TIME^[109]^. Hyperactivation of focal adhesion kinase (FAK) (also known as FADK) in tumor cells induces overexpression of various chemokines (including CCL5), thus recruiting Treg cells and inducing CD8+ T cell exclusion or exhaustion. Self-antigen-specific CD8+ T cells express restricted co-stimulatory signals, which impair APC activity and enhance suppression by Tregs, whereas Tregs have a stronger affinity for TCRs of non-self-antigen specific CD8+ T cells, which are resistant to suppression^[121,122]^.

Immune checkpoints like CTLA-4 and PD-1 are expressed by Tregs as well. In human glioblastoma tissue, Tregs with high PD-1 exhibit an exhausted phenotype lacking immunosuppressive activity. Anti-PD-1 mAbs may cause hyper-progression, decreasing the number of Tregs that express PD-1 and restore their ability to inhibit immunological response^[121,123-125]^.

The contribution of the B7x immunological checkpoint to the growth of the Treg population within the tumor was discussed by John *et al*. In accordance with their suppressive function, B7x+ Tregs exhibited a higher level of TGF-LAP (a surface marker of TGF production) and a lower percentage of ki67 marker, indicating that they originated from peripherally converted CD4+ T cells. B7x increased Foxp3 expression through increasing STAT5 phosphorylation, whereas it inhibited STAT3 phosphorylation, which affected CD4+ T cells’ ability to differentiate into the Th17 subtype. What is more intriguing is that the anti-CTLA-4 therapy was not successful in reducing Tregs exclusively in mice that expressed B7x+. In addition, B7x+ mice showed a substantial reduction in the IFN production generally caused by anti-CTLA-4. Tregs play a crucial part in the immune response, as shown by the fact that Treg depletion restored anti-CTLA-4 capabilities in tumor reduction even in B7x+ mice^[126]^.

#### MDSCs

MDSCs may differentiate into M2-polarized macrophages with immunosuppressive properties and can decrease T-cell activation by metabolic processes (e.g., iNOS, IDO). Additionally, they are able to express CD40 and to produce TGF- and IL-1, leading to Tregs expansion and decreasing the capacity of effector T cells^[93]^. In both melanoma patients and prostate cancer patients treated with ipilimumab, a lower baseline amount of circulating MDSCs was associated with a higher overall survival rate. Furthermore, high myeloid inflammation gene signature expression was associated with reduced PFS in the atezolizumab monotherapy arm and in the atezolizumab + bevacizumab arm, but not in the sunitinib arm in the molecular analysis of the IMmotion 150^[127,22]^.

#### DCs

According to their presence in Tertiary lymphoid structures (TLS), DCs in ccRCC can be divided into two subtypes: TLS-DCs (CD83+ DC- LAMP+), which are very uncommon in ccRCC and associated with good outcomes, and non-TLS-DCs (CD209+ CD83), which are dominant in ccRCC TIME and associated with the worst prognosis. In addition to promoting tumor growth by secreting MMP-9 and TNF, NTLS-DCs further inhibit CD8+ T-cell activity by the L-arginine pathway and trigger Treg responses by secreting TGF-^[109,119,128,129]^.

### Genomic and single-gene mutation and TIME

The biomarker analyses conducted among patients treated in the IMmotion 150, the Javelin Renal 101 and the Checkmate 214 had developed the idea that gene expression profile (GEP) in RCC could predict response to ICIs^[22-24]^.

Based on mRNA expression data, Beuselinck *et al.* performed a clustering analysis and identified four molecular subgroups of ccRCC, even if done in a cohort of patients receiving sunitinib: ccrcc1 (“c-myc-up”), ccrcc2 tumors (“classical”), ccrcc3 (“normal-like”) and ccrcc4 tumors (“c-myc-up and immune-up”). The ccRCC4 subtype displayed a strong inflammatory, Th1-oriented but suppressive immune microenvironment, with a strong expression of myeloid and T-cell homing factors. Furthermore, this subtype had a higher proportion of IL10 as well as inhibitory receptors LAG3 and PD-1 and PD-1 ligands PD-L1 and PD-L2. In the validation analysis of single-gene mutations conducted on TGCA samples, SETD2 mutations were related to a lower T-cell infiltration and immunosuppressive markers (ccrcc1), while BAP1 mutations were expressed in the subtype with the highest inflammatory infiltration but on the other hand, having the strongest expression of immunosuppressive cells (ccrcc4)^[130]^.

In the BIONIKK trial, the authors used Beuselinck’s clustering classification to undertake a biomarker-driven analysis. Patients with the ccrcc 1 and 4 subtypes were randomized to receive nivolumab either alone or in combination with ipilimumab, whereas those with ccrcc 2 and 3 could receive either nivolumab plus ipilimumab or a VEGF-TKI. In the ccrcc1 (immune desert subtype), ORR and PFS were improved by the dual checkpoint inhibition (HR of PFS for nivolumab *vs*. nivolumab + ipilimumab 1.27; 95%CI 0.77-2.11). In the ccrcc4 (immune infiltrated and inflammatory subtype), both nivolumab alone and in combination with ipilimumab obtained higher ORR and longer PFS compared to the ccrcc 1 group. Thus, ccrcc4 seemed the best candidate for dual checkpoint inhibition. Furthermore, about 30% of patients in the ccrcc4 group who early progressed on nivolumab–ipilimumab did not start a second-line therapy, thus reflecting that progression at first evaluation, does not always indicate resistance^[131]^.

#### PBRM1 and BAP1

PBRM1 encodes for BAF180, a component of the SWI/SNF chromatin remodeling complex and could be inactivated in about 36% of clear cell renal cell carcinoma. Its relationship with prognosis in RCC has been evaluated in several studies, resulting in conflicting findings. PBRM1 mutations have also been documented in VHL-disease-associated RCC. PBRM1 and VHL mutations were most frequently expressed in ccrcc1 subtypes (immune desert one)^[131-134]^. In the phase I CA209-009, 35 samples of RCC treated with Nivolumab were whole-exome sequenced and were consequently divided into three categories according to responses. Clinical response to Nivolumab was characterized by a higher percentage of PBRM1 loss-of-function. The same results were seen in a subgroup treated with nivolumab plus ipilimumab^[135]^.

As reported above, in the molecular subsets analysis of the IMmotion 151, PBRM1 mutations conferred better outcomes to patients, regardless of the treatment arm. However, in patients with PBRM1-non-mutant tumors, the addiction of immunotherapy to a VEGFR-TKI confers better outcomes compared to target therapy alone^[31]^.

#### SETD2

SETD2 mutations, a histone methyltransferase gene, occur in 10% of ccRCC. Wang *et al.* clustered TGCA RCC samples based on TME expression profiles and observed two different clusters: the first cluster, inflamed subtype (IS), was enriched for Treg cells, NK cells, Th cells, neutrophils, macrophages, eosinophils, B cells and CD8+ T cells, whereas the second cluster, not inflamed subtype (NIS), was enriched for angiogenesis, plasmacytoid DCs, and mast cells. Mutations in BAP1 were most frequently seen in the IS (*P* = 7.7 × 10^-5^), whereas the NIS was enriched for PBRM1 mutations. Furthermore, the authors observed that Bap1-mutated mice were more infiltrated by CD4 and CD8 T cells, than Pbrm1-mutated mice, suggesting a causal relationship between BAP1 mutations and the TME-IS phenotype. It is interesting to note that individuals with tumors of the TME-IS subtype had higher rates of thrombocytosis and anemia, which are systemic symptoms of inflammation caused in TME^[136]^.

## GUT MICROBIOME INFLUENCES PRIMARY RESISTANCE TO IMMUNOTHERAPY

The complex system of the gut microbiota interacts with the host. By producing neoantigens and modifying the tumor immunological microenvironment, microorganisms have an impact on immune responses. Furthermore, studies have revealed that antibiotics can affect the metabolic balance of the intestinal microbiome by increasing some species while decreasing others, leading to dysbiosis^[137-140]^.

Routy *et al.* demonstrated that the use of antibiotics (ATB) influenced response to ICIs in non-small cell lung cancer (NSCLC), RCC and urothelial cancers. As evidenced by poorer outcomes for individuals receiving antibiotic treatment, ATB usage did, in fact, cause resistance to PD-1 blockade in all tumor types. Analyzing the microbiome composition, *Akkermansia muciniphila* conferred better PFS to patients treated with immunotherapy. Th1 and Tc1 cell reactivity against *A. muciniphila* was the only immune response to ICIs that was associated with clinical outcomes^[138]^.

De rosa *et al*. confirmed that ATB impaired patients’ responses to immunotherapy by altering the diversity and composition of gut microbiota in a metagenomic and network analysis of patients treated with nivolumab in the NIVOREN GETUG-AFU 26 phase 2 trial. Responders had an over-representation of distinct species including *A. muciniphila, Bacteroides salyersiae*, and *Eubacterium siraeum*, and a trend towards *Clostridium ramosum* and *Alistipes senegalensis*, whereas *E. bacterium*_2_2_44A, *Clostridium hathewayi*, and *Clostridium clostridioforme* were more represented in non-responders, as observed for those patients using ATB. Due to the fact that the majority of patients had already received a VEGF-TKI, the authors examined the effects of TKI use in combination with ATB on microbiome composition and discovered that axitinib + ATB was the most effective treatment to induce a shift in fecal microbiota. Additionally, most notably with cabozantinib, TKIs promoted a transition to a preponderance of immunostimulatory commensals, such as A. senegalensis and A. muciniphila. This property could be one of the rational bases for combining VEGFR-TKI with ICIs^[141]^.

## ACQUIRED RESISTANCE TO IMMUNE CHECKPOINT INHIBITORS

Secondary resistance develops throughout therapy and may be driven by the stress that a particular therapy might have on TIME, as well as by temporal variability and plasticity. The same mechanisms that lead to initial resistance also cause secondary resistance to immune-checkpoint inhibitors. Some investigators observed that TILs (the immune response’s effectors) are present during relapse but remain restricted to the tumor margin, raising the possibility that these cells have lost the ability to identify antigens or to activate themselves. Utilizing ICIs, the IFN-induced expression of PD-L1 and its negative effects on CD8+ T cells are prevented. To reduce antigen presentation or enable escape from interferon-induced growth inhibition, cancer cells may, however, become insensitive to IFN signaling. JAK mutations have been investigated as a strategy by which cancers develop IFN unresponsiveness^[142]^.

Numerous investigations conducted on humans have shown that tumor cells can develop resistance to T-cell recognition by having defective HLA class I expression^[142-145]^. Loss-of-function in chromosome region 15q21-(where β2m gene maps) induces a *β2m* gene mutation, leading to the absence of the MHC class I. These alterations can be reversible and the HLA expression can be recovered by using immunotherapy, as a result of transcriptional silencing of genes or irreversible. It has been suggested that resistance to immunotherapy may be caused by the preexistence of metastatic lesions with a *β2m* gene mutation and that selective pressure during T-cell-based immunotherapy could induce the growth of HLA class-I deficient clones in melanoma patients who have irreversible HLA alterations^[146]^.

Chronically activated Treg cells showed strong suppressive potential. Tregs increase CD103 expression when activated, and CD103+ Treg exhibited greater levels of inhibitory receptors such as PD-1, TIM-3, and CTLA-4. Additionally, they upregulate functional molecules, granzyme B and IL-10, which are essential to their suppressive actions, such as the decrease in CD8+ T cells^[147]^.

## ALTERNATIVE INHIBITORY RECEPTORS UPREGULATION AS A MECHANISM OF ACQUIRED RESISTANCE

Exhausted CD8+ T cells lose their cytotoxic efficacy against presented antigens. PD-1 is only one of the inhibitory receptors highly expressed on exhausted CD8 T cells and its blockade may have a therapeutical effect. However, blocking this pathway does not completely restore T cell function. Numerous alternative inhibitory receptors, primarily identified in chronic infections, such as LAG-3, TIM-3, 2B4, CD160, or BTLA, are overexpressed in exhausted T cells and may be targeted to enhance immune response or reverse resistance^[147,148]^.

### PD-1

PD-1 is an inhibitor of both adaptive and innate immune responses, and it is expressed on activated T, natural killer (NK) and B lymphocytes, dendritic cells (DCs), macrophages, and monocytes^[149]^. In a cohort of patients with NSCLC, gastric cancer (GC) and melanoma treated with Nivolumab, CD8+ T cells of the TIMEs from responders expressed higher levels of PD-1 than those from non-responders. Furthermore, PD-1 was highly expressed in eTreg (CD45RA−CD25^hi^Foxp3^hi^CD4+) of the TIME of patients with NSCLC and GC which not responded to nivolumab. In this context, the use of aPD-1 mAb could have immunosuppressive effects, enhancing the expression of PD-1 among eTreg, and thus stimulating Treg functions^[150]^.

PD-1 expression balance between CD8+ T cells and Treg cells is crucial for the efficacy of monoclonal antibodies to PD-1.

The prognostic value of PD-L1 expression in RCC is controversial. A meta-analysis of four trials assessed the predictive value of PD-L1 among patients treated with nivolumab + ipilimumab, atezolizumab + bevacizumab, pembrolizumab + axitinib or avelumab + axitinib *vs.* sunitinib. Patients with PD-L1-positive tumors showed significantly improved ORRs, complete response rates (CRRs) and PFS, and lower progression disease responses (PDRs) compared with those with PD-L1-negative tumors when treated with ICIs *vs.* sunitinib. Nivolumab plus ipilimumab had the highest likelihood of providing the maximal PFS (p score: 0.90) and the highest ORR (p score: 0.95)^[151]^.

Overall, even though the findings of this study show that PD-L1 has a predictive value, there are several limitations, such as the fact that PD-L1 is expressed differently in primary tumors compared to metastatic sites, as well as the lack of a standard detection method and the different types of anti-PD-L1 antibody used. Examining the variable pattern of PD-L1 expression on different immune cells in TIME might be more fascinating than examining its expression just on tumor cells. As reported, it appears that the effectiveness of ICIs depends on the balance between PD-1 expression on CD8+ T-cells and Treg.

### LAG3

Lymphocyte Activation Gene-3 (LAG3; CD223) is expressed on activated human NK and T cell lines and is able to bind MHC class II. A soluble monomeric form of LAG3 (sLAG3) can be released by IFN-producing CD4+ T cells. The major ligand of LAG3 is MHC class II. Melanoma cells that express MHC class II attract tumor-specific CD4+ T cells through their interaction with LAG3, resulting in impairing CD8+ T cell responses. Other putative ligands are Galectin-3, which mediates suppression of CD8+ T-cell-secreted IFN in vitro and LSECtin (liver sinusoidal endothelial cell lectin). It was found that the association between LAG3 and the LSECtin ligand inhibits the generation of IFN by effector T cells that are antigen-specific in melanoma cells. Dendritic cell inhibition is one of the ways whereby LAG3-expressing Tregs interact with MHC class II to cause immunological suppression. In fact, when exposed to LAG-3, MHC class II-expressing melanoma cells, but not MHC class II-negative ones, were resistant to Fas-mediated apoptosis^[152-155]^.

In a study examining the key inhibitory receptors (iR) expression on TILs and PBMCs of 35 patients with RCC, CD8+ T cells and non-Treg CD4+ cells highly expressed the inhibitory receptors PD-1, followed by LAG-3 and BTLA, whereas Tim-3 and CTLA-4 were less highly expressed. However, the expression profile of the five iR on tumor-infiltrating Tregs was different. Indeed, Tregs upregulated PD-1, LAG-3, Tim-3, and CTLA-4, but not BTLA. Interestingly, as previously reported, the most frequent iR combination was PD-1 and LAG-3, whereas about 10% of CD8+ T cells expressed PD-1, LAG-3 and Tim-3 simultaneously. Experimental in vitro demonstrated that blockade of PD-1 plus LAG-3 resulted in a statistically significant higher percentage of CD8+ IFN+ T cells, than blockade of PD-1 alone.

Additionally, when CD4+ and CD8+ T cells were co-cultured with anti-PD-1, LAG3 upregulated but not PD-1. These findings suggested that blocking LAG-3 in combination with PD-1 might be an effective treatment for advanced RCC^[156]^.

### TIM-3

T cell immunoglobulin and mucin-domain containing-3 (Tim-3) is a type I trans membrane protein expressed in IFN-γ-producing Th1 and Tc1 cells. The expression of cytokines such as TNF and INF-γ and Th1 responses are significantly suppressed by Tim-3. Tim-3 is linked to T cell exhaustion and its expression on CD8+ T cells is directly related to PD-1. Indeed, CD8+ T cells co-expressing Tim-3 and PD-1 are “deeply” exhausted T cells. Tim-3 could be expressed on tumor-infiltrating DCs, playing a role as a mediator of the innate immune response. The Tim-3 ligand with the highest affinity is galectin-9, and its interaction with Tim-3 causes the death of effector Th1 cells and CD8+ T cells. Furthermore, galectin-9 increases Tim-3-mediated IFN production in NK cells, activates PI3K-mTOR signaling in myeloid cells, and alters cytokine production by monocytes/macrophages, affecting Th1 and Th17 responses.

Other theorized ligands are being researched. Galectin-9 and Ceacam1 collaborate, both having a comparable effect. Activated DCs release the damage-associated molecular pattern known as HMGB1 (high-mobility group box 1), which stimulates T and B cell responses while inhibiting innate immune responses to tumor-derived nucleic acids. Tim-3 signaling’s impact is influenced by the ligands involved, the cellular environment, and the biological state, particularly whether the stimulation is acute or persistent. Tim-3 can have adjuvant effects inducing the expression of co-stimulatory receptors. In contrast, chronic stimulation could enhance the inhibitory functions of Tim-3 signaling, especially as concerns HLA-B associated transcript 3 (Bat3) deficient Th1 and CD8+ T cells, driving these cells to an exhaustion stage^[157-164]^.

Expression of Tim-3 on T cells also plays a critical role in the generation of MDSCs and is even present in FoxP3+ T regs, contributing to promoting T cell dysfunction and immune suppression^[165,166]^.

In isolated CD8+ T cells, Tim-3+PD-1+ TILs were identified as an exhausted phenotype of T cells, having a reduced production of IL-2, TNF, and IFN-γ. Combining anti-Tim-3 and anti-PD-L1 therapy reduced the tumor growth in mouse models, and those who underwent a complete regression continued to be tumor-free even after rechallenging^[167]^.

Tim-3 expression in RCC has been associated with outcomes resulting in contradictory results. Patients receiving nivolumab were assessed in the CheckMate-010 research study conducted by Pignon *et al*. in anattempt to identify the mechanisms causing different responses. Longer median immune-related response progression-free survival (irPFS) and higher ORR were associated with the presence of CD8+ tumor-infiltrating cells that express PD-1 but lack LAG3 and TIM3 and are more likely to be T cell activated. This is in contrast to Zelba *et al*.’s findings, which showed that blocking both PD-1 and Tim-3 simultaneously had no effect on the average cytokine production by TILs, resulting in a lack of the immune response’s restoration. Therefore, additional research is required to confirm the usefulness of Tim-3 as a possible target to enhance immune responses driven by the inhibition of checkpoint inhibitors^[168,156]^ [Table 4].

**Table 4:** Major determinants of primary and acquired resistance to ICIs

**Primary resistance to ICIs**	
Antigen availability	mRCC has a low mutation load
Antigen presentation defects	Beta-2 microglobulin loss might result in a deficiency of MHC class I expression.
Absence of dendritic cells (DCs)	Due to defects in chemotactic signals, active -catenin signaling causes the depletion of DCs.
Antigen epigenetic modification	hHERVs may influence both innate and adaptative immune responses.
Cells trafficking and recruitment alterations	·WNT/-catenin pathway activation reduces DCs recruitment. ·PTEN loss reduces CD8+T cell infiltration.
T-cell activity inhibition	JAK1/2 loss of function mutations inhibit T cell killing efficacy and IFN-mediated PD-L1 expression.
Tumor microenvironment composition	TAMs ·M2-like TAMs correlate with worse prognosis in mRCC Tregs ·Anti-CTLA-4 mAbs are not effective in depleting Tregs expressing B7x immune checkpoint. MDSCs ·Low MDSCs infiltration correlates with better outcomes with ICIs DCs ·A specific subtype of DCs (NTLS-DCs) with immunosuppressive functions is dominant in ccRCC.
Genomic and single-gene mutations	PBRM1 ·Regardless of the type of treatment utilized, PBRM1 mutations are correlated with better results. ·Combining VEGFR-TKI with ICI is more effective in PBRM1 non-mutant tumors. ·PBRM1 mutations are more frequent in non-inflamed, angiogenic subtypes. SETD2 ·SETD2 mutations are enriched in inflamed immune infiltrated subtypes.
Gut microbiome composition	Antibiotic use induces resistance to aPD-L1 mAb Different bacterial species are represented in responders *vs.* non-responders
**Secondary resistance to ICIs**	
Same mechanisms involved in primary resistance	·IFN unresponsiveness via JAK mutations ·HLA I defects. ·Loss-of-function of Beta-2 microglobulin mutations.
Alternative inhibitory receptors	·LAG3 ·TIM-3

DCs: Dendritic cells; hHERVs: human endogenous retroviruses; HLA: human leukocyte antigen; IFN, interferon; JAK: janus kinases; LAG3: lymphocyte activation gene-3; MDSCs: myeloid-derived suppressor cells; mRCC: metastatic renal cell carcinoma; PD-L1: programmed death-ligand 1; TAMs: tumor-associated macrophages; TIM-3: T-cell immunoglobulin and mucin-domain containing-3; Tregs: T regulator.

## CONCLUSIONS

Metastatic renal cell carcinoma management has undergone a paradigm shift as a result of the development of combination therapy using ICIs. The choice of first-line treatment and its correct application continues to be crucial factors in tumor evolution and have the potential to cause initial resistance, which may have an impact on overall survival. Because of this, it is now crucial to understand how the combination of ICIs or the addition of a VEGF-TKI to immunotherapy may alter the tumor microenvironment and affect the tumor’s response to treatment. Targeting hypoxia with specific drugs may be a possibility to enhance outcomes in RCC since hypoxia is a defining feature of RCC pathogenesis and is associated with both angiogenesis and the alteration of the tumor microenvironment. HIF-2 inhibitor belzutifan is being tested in patients with previously treated mRCC as a single treatment versus everolimus (NCT04195750) or in combination with lenvatinib versus cabozantinib (NCT04586231). Different inhibitory receptors or metabolic pathways may be the focus of alternative approaches. The phase II trial FRACTION-RCC is studying an anti-LAG3 mAb (relatlimab), in combination with nivolumab (NCT02996110). XmAb®22,841, a bispecific antibody targeting CTLA-4 and LAG-3, is under study in the phase I trial DUET-4 (NCT03849469). A randomized phase II trial is investigating the efficacy of axitinib combined with an antibody against OX40, a receptor expressed on memory T cells (NCT03092856). In a phase 1b/2 trial, patients with previously treated mRCC are being treated with cabozantinib in conjunction with the anti-AXL fusion protein AVB-S6-500, which controls the GAS6/AXL signaling pathway (NCT04300140).

Despite the previously mentioned advancements, there is still a significant research gap in the individual biology of tumors. Furthermore, there is no trustworthy biomarker to direct patient selection. The dynamic genomic and immunomodulatory alterations that systemic treatment causes in the TIME in advanced ccRCC may help to partially explain this paucity of biomarkers.

It may be possible to predict therapy response by combining tumor genomic and immune signatures, but much more accurate research is needed to link biological understanding with clinical findings in RCC.

We must change how we approach treating ccRCC due to the advent of clinical resistance to the currently available systemic treatments. Cancer cells are constantly changing as a result of therapy pressure, plasticity, and heterogeneity. We may be able to comprehend, avoid, and overcome resistance mechanisms by estimating the trajectory of ccRCC evolution. In the prospective cohort study TRACERx, the authors conducted a whole genome sequencing of RCC tumor samples to generate information on the timing of driver mutations, level of intratumoral heterogeneity, and presence of parallel evolution in each patient. They found that the loss of heterozygosis of chromosome 3p was the first critical driving event. The most frequent alteration was rearrangement between 3p and 5q (one copy of 3p lost and one copy of 5q earned), defined t (3:5) chromothripsis. Using t (3:5) as the cut-off, they calculated the chronological age at which each mutation occurred. They found that the duplication that caused t (3:5) chromothripsis was an early event (35-50 years before tumor diagnosis), causing a modest initial clonal expansion, and that the mutation rate throughout life remained constant. Due to the latency between the triggering mutational event and subsequent progression, there may be a window for early intervention to prevent RCC. Indeed, the incidence of sporadic RCC could be decreased by reducing the 3p-LOH clone size by 50%. This is reasonable given that this chromosome contains four tumor-suppressor genes (VHL, PBRM1, BAP1, and SETD2) that are crucial for cellular survival^[169]^.

Based on seven evolutionary subgroups that coincide with clinical characteristics, ccRCCs have been divided into four groups^[169,170]^. These groups are distinguished by four features-variations in chromosomal complexity, intra-tumor heterogeneity (ITH), model of tumor evolution, and metastatic potential.

In a review by Kowalewski *et al.*, the authors investigate single group characteristics and describe possible evolution-target strategies according to the evolutionary trajectories. Group 1 tumors are those that have a single VHL mutation and a low genome instability index (wGII), as well as low ITH. Given the positive predictive significance of a low wGII as a measure of response to ICIs, this group may benefit from immunotherapy. Furthermore, a stable tumor burden could be reinforced by adaptative therapy, upfront cytoreductive nephrectomy (CN), and treatment targeting trunk group before the loss of 9p or 14q, which marks the acquisition of metastatic competence. Group 2 tumors are those with an early PBRM1 mutation and a subsequent SETD2 mutation, PI3K pathway mutation, or high wGII, and were distinguished by a “branched” evolutionary pattern. In this group, modulating genomic instability could be useless, whereas targeting immune evasion could be an option. In contrast, Group 3 and 4 tumors are those with multiple driver mutations (VHL plus ≥ 2 BAP1, PBRM1, SETD2, or PTEN) resulting in “punctuated” evolution and characterized by high wGII. ITH is low in group 3 tumors but higher in group 4 tumors, giving that group a rapid dissemination pattern. Due to high wGII and a punctuated evolution pattern in Groups 3 and 4, it may be effective to address genomic instability by enhancing it. The goal of evolutionary herding is to reduce ITH and manage any potential distinct clones that may result from a prior treatment by utilizing a combination of drugs in a specific order. Hence, it should be considered in Groups 1 and 3^[171]^.

We might be able to transpose this perspective into the real world with the aid of new technologies. For instance, the repeated evolution in cancer (REVOLVER) machine-learning algorithm was created to achieve repeatable disease prognosis based on next-generation sequencing (NGS) count data, thereby classifying patients based on the evolution of their tumors over time^[172]^. Trials including biomarkers and evolutionary paths as drivers of chosen treatment will be the new challenge in the future, in order to predict earlier the correct strategy and to prevent a manipulation that could be harmful when applied in the incorrect evolutionary trajectory.

## DECLARATIONS

### Copyright

© The Author(s) 2023.
